# Hemimethylation of CpG dyads is characteristic of secondary DMRs associated with imprinted loci and correlates with 5-hydroxymethylcytosine at paternally methylated sequences

**DOI:** 10.1186/s13072-019-0309-2

**Published:** 2019-10-17

**Authors:** Julianna Nechin, Emma Tunstall, Naideline Raymond, Nicole Hamagami, Chris Pathmanabhan, Samantha Forestier, Tamara L. Davis

**Affiliations:** Department of Biology, Bryn Mawr College, 101 N. Merion Avenue, Bryn Mawr, PA 19010-2899 USA

**Keywords:** Genomic imprinting, DNA methylation, *H19*, *Cdkn1c*, *Snrpn*, *Ndn*, *Peg12*, Secondary DMR, Epigenetics

## Abstract

**Background:**

In mammals, the regulation of imprinted genes is controlled by differential methylation at imprinting control regions which acquire parent of origin-specific methylation patterns during gametogenesis and retain differences in allelic methylation status throughout fertilization and subsequent somatic cell divisions. In addition, many imprinted genes acquire differential methylation during post-implantation development; these secondary differentially methylated regions appear necessary to maintain the imprinted expression state of individual genes. Despite the requirement for both types of differentially methylated sequence elements to achieve proper expression across imprinting clusters, methylation patterns are more labile at secondary differentially methylated regions. To understand the nature of this variability, we analyzed CpG dyad methylation patterns at both paternally and maternally methylated imprinted loci within multiple imprinting clusters.

**Results:**

We determined that both paternally and maternally methylated secondary differentially methylated regions associated with imprinted genes display high levels of hemimethylation, 29–49%, in comparison to imprinting control regions which exhibited 8–12% hemimethylation. To explore how hemimethylation could arise, we assessed the differentially methylated regions for the presence of 5-hydroxymethylcytosine which could cause methylation to be lost via either passive and/or active demethylation mechanisms. We found enrichment of 5-hydroxymethylcytosine at paternally methylated secondary differentially methylated regions, but not at the maternally methylated sites we analyzed in this study.

**Conclusions:**

We found high levels of hemimethylation to be a generalizable characteristic of secondary differentially methylated regions associated with imprinted genes. We propose that 5-hydroxymethylcytosine enrichment may be responsible for the variability in methylation status at paternally methylated secondary differentially methylated regions associated with imprinted genes. We further suggest that the high incidence of hemimethylation at secondary differentially methylated regions must be counteracted by continuous methylation acquisition at these loci.

## Background

Genomic imprinting refers to the parent of origin-specific expression of one parental allele over another. To date, approximately 150 mammalian genes have been found to exhibit this unusual form of regulation [1, 2]. Parent of origin-specific expression of imprinted genes is achieved via multiple mechanisms, including differential DNA methylation, differential distribution of modified histones and differential expression of long non-coding RNAs from the maternal vs. paternal alleles [3, 4]. Importantly, all imprinted genes are associated with an imprinting control region, which is differentially methylated on the parental alleles and is responsible for the regulation of the genes located through the associated imprinting cluster [3].

Differential DNA methylation associated with imprinted genes can be categorized into two classes. One class is comprised of primary or gametic differentially methylated regions (DMRs), at which the differentially methylated state associated with the parental alleles is acquired during gametogenesis, inherited at fertilization, and maintained throughout development, including during the genome-wide demethylation that occurs prior to implantation [3]. These primary DMRs typically correlate with the imprinting control region and play a critical role in the establishment and maintenance of imprinted gene expression by affecting the activity of insulators or the expression of long non-coding RNAs that regulate adjacent imprinted genes [5–8]. In addition, some primary DMRs directly affect the expression of protein-coding imprinted genes via differential methylation of their promoters [9]. In contrast, secondary DMRs acquire their differentially methylated state during post-implantation development [10–14]. The role of secondary DMRs is less clear, though evidence suggests they may be important for maintaining imprinted expression as they are typically located at promoters and failure to establish or maintain methylation at these loci results in the dysregulation of the associated imprinted gene [12, 15–18].

Previous work has illustrated that there is variation in the stability of DNA methylation at primary vs. secondary DMRs associated with imprinted genes. Primary DMRs typically display very high levels of DNA methylation on the methylated allele (90–100%) and very low levels of DNA methylation on the unmethylated allele (0–10%) [11, 14, 19–22]. In contrast, significantly more variations in DNA methylation patterns are observed at secondary DMRs. The methylated allele typically has less consistent DNA methylation than is observed at primary DMRs [10, 11, 13, 23]. In addition, some secondary DMRs display DNA methylation on both parental alleles, although one allele contains significantly more methylation than the other [24]. Investigation into the more variably methylated secondary DMRs has shown that substantial levels of asymmetric DNA methylation are observed at CpG dyads. For example, the variably methylated secondary DMRs associated with the imprinted *Dlk1* and *Gtl2* genes contain 29% and 32% hemimethylation, as compared to 8% hemimethylation at the primary IG-DMR associated with the *Dlk1*/*Gtl2* imprinting cluster on mouse chromosome 12 [13, 24]. The high level of methylation asymmetry observed at these secondary DMRs explains the variability in the DNA methylation patterns and could be a consequence of TET activity at these loci, which could lead to the active demethylation of cytosines in these regions and could also result in passive DNA demethylation via the reduced activity of Dnmt1 at oxidized methylcytosine [25–29].

**Fig. 1:**
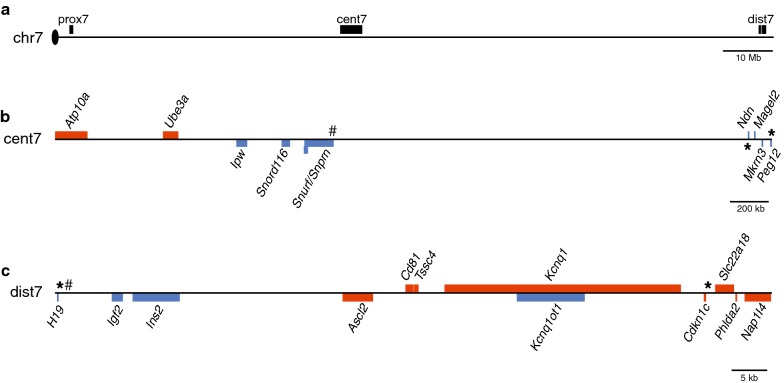
Imprinting clusters on mouse chromosome 7. **a** Location of proximal, central and distal imprinting clusters. Detail of central (**b**) and distal (**c**) imprinting clusters. Red and blue rectangles correspond to maternally and paternally expressed genes, respectively. Genes located above and below the line have + and − strand orientation, respectively. Pound signs and asterisks indicate the location, respectively, of primary and secondary DMRs analyzed in this study. *Snrpn*, maternally methylated primary DMR; *Ndn* and *Peg12*, maternally methylated secondary DMRs; *H19* ICR, paternally methylated primary DMR; *H19*-pp and *Cdkn1c*, paternally methylated secondary DMRs

To determine if high levels of hemimethylation are characteristic of secondary DMRs associated with imprinted loci, we investigated CpG dyad methylation patterns at well-characterized paternally and maternally methylated primary and secondary DMRs located in the central and distal imprinting clusters on mouse chromosome 7; we did not analyze loci in the proximal imprinting cluster (Fig. 1). We further examined the correlation between hemimethylation levels and the presence of 5-hydroxymethylcytosine (5hmC) to test the hypothesis that oxidation and removal of methylcytosine is responsible for the variable DNA methylation patterns at these loci. Finally, we examined the sequence composition at primary vs. secondary paternal and maternal DMRs to test the hypothesis that sequence context may play a role in the difference in DNA methylation stability associated with primary vs. secondary DMRs. Briefly, we consistently observed high levels of hemimethylation at secondary DMRs, regardless of which parental allele was methylated, and high levels of hemimethylation were correlated with the presence of 5hmC at paternally methylated sequences.

## Results

### CpG dyads within paternally and maternally methylated secondary DMRs associated with imprinted genes display high levels of hemimethylation

Previous work in our lab illustrated a high level of hemimethylation at two paternally methylated secondary DMRs located in the *Dlk1*/*Gtl2* imprinting cluster on mouse chromosome 12. To assess whether hemimethylation is generally a feature of secondary DMRs or whether this phenomenon is unique to loci in the *Dlk1*/*Gtl2* imprinting cluster, we examined DNA methylation at CpG dyads located within the paternally methylated secondary DMRs associated with *H19* and *Cdkn1c*; these DMRs are located in two different imprinting clusters located on mouse chromosome 7 (Fig. 1, [11, 12]). We assessed methylation at various stages of development to assess both the establishment of differential methylation and its maintenance; in general, levels of methylation did not vary significantly during development indicating that overall levels of methylation remain similar over time (Additional file 1). All analyses were conducted using F_1_ hybrid mice to utilize C57BL/6J vs. *Mus musculus castaneus* SNPs to distinguish parental origin of each allele (see “Methods”).

**Fig. 2:**
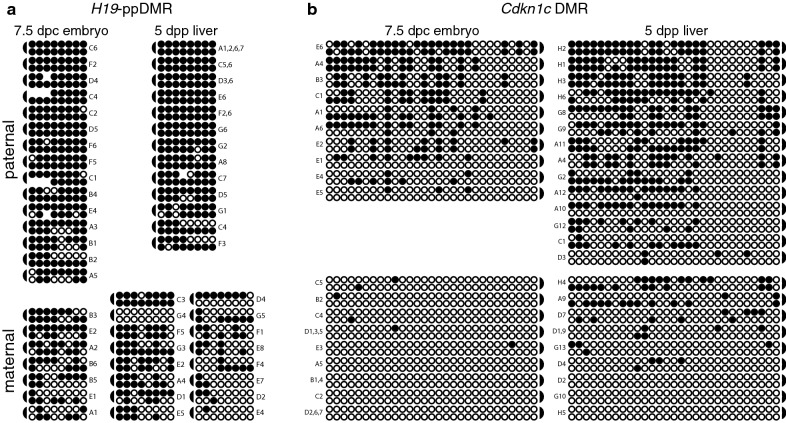
The paternally methylated secondary DMRs associated with *H19* and *Cdkn1c* display high levels of hemimethylation. Bisulfite mutagenesis and sequencing of F_1_ hybrid DNA derived from 7.5 dpc BxC embryos and 5 dpp BxC liver. Individual circles in each row represent one of the potentially methylated CpG dinucleotides analyzed at the *H19*-ppDMR (**a**) or the *Cdkn1c* DMR (**b**), and each paired row of circles represents the complementary strands of an individual subclone; semi-circles to the right or left indicate the location of the linker connecting the complementary strands. Filled circles represent methylated cytosines, open circles represent unmethylated cytosines, absent circles represent ambiguous data. Alphanumeric labels identify subclones analyzed; letters represent independent amplification reactions, while numbers represent individual subclones. Subclones derived from the same amplification that have identical sequence and methylation patterns are grouped together, as it was not possible to determine if these amplicons were derived from the same or different template molecules. Data obtained from 14.5 dpc BxC embryos and adult BxC liver are shown in Additional file 7: Figure S1. Reciprocal cross-data obtained from 13.5 dpc CxB embryos are shown in Additional file 10: Figure S4

At *H19*, we analyzed 8 CpG dyads that had previously been shown to be part of the promoter-proximal *H19* secondary DMR (*H19*-ppDMR) [11]. Of note, in all the tissues we analyzed, we detected significantly more DNA methylation on both the paternal and the maternal alleles than had been observed previously ([11]; Fig. 2a). This difference may be attributed to differences in genetic background. Despite the higher levels of methylation we observed overall in this region, we determined that the amount of methylation on the paternal alleles was significantly higher than on the maternal alleles throughout development using a Mann–Whitney *U* test (*P* = 0.0012, 7.5 dpc embryo; 0.0477, 14.5 dpc embryo; 0.0001, 5 dpp liver; 0.0008, adult liver), indicating that this region was differentially methylated in the F_1_ hybrid mice used in our study. In addition, Mann–Whitney *U* tests indicated that methylation levels remained constant on the parental alleles across development (data not shown). Hemimethylation at the *H19* secondary DMR averaged 31.9% for both parental alleles across development (Fig. 2a, Additional file 2). Using a Chi square test of independence, we found that the level of hemimethylation at the *H19* secondary DMR was not significantly different than the levels we had previously observed at the *Dlk1*- and *Gtl2*-DMRs (*P *= 0.2231 and 0.8370, respectively).

We also analyzed 29 CpG dyads located at the 5′ end of the *Cdkn1c* DMR analyzed by Bhogal et al. [12]. *Cdkn1c* displayed even higher levels of hemimethylation than we detected at *H19*. Methylation levels on each parental allele remained constant across development, averaging 48.5% for both parental alleles (Fig. 2b, Additional file 2). The level of hemimethylation at the *Cdkn1c* DMR was significantly higher than the level observed at the *Gtl2, Dlk1* or *H19* secondary DMRs (*P *= 2.45 × 10^−7^, 1.39 × 10^−14^ and 1.31 × 10^−15^, respectively).

Similar to paternally methylated secondary DMRs, maternally methylated secondary DMRs exhibit more variable DNA methylation patterns than primary DMRs associated with imprinted loci [10, 30–32]. Based on this similarity, we hypothesized that maternally methylated secondary DMRs would also exhibit high levels of hemimethylation. We therefore examined DNA methylation at CpG dyads located within the maternally methylated secondary DMRs associated with *Ndn* and *Peg12* on mouse chromosome 7 [10, 30].

**Fig. 3:**
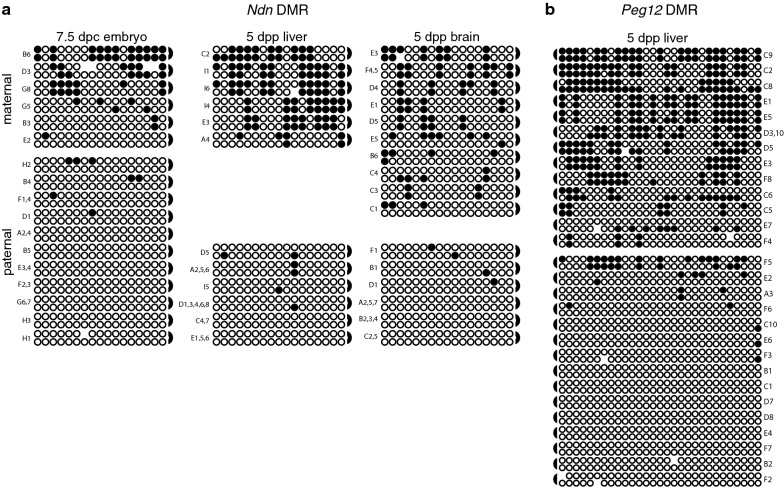
The maternally methylated secondary DMRs associated with *Ndn* and *Peg12* display high levels of hemimethylation. **a** Methylation status at the *Ndn* DMR; F_1_ hybrid DNA derived from 7.5 dpc BxC embryos and 5 dpp BxC liver and brain. **b** Methylation status at the *Peg12* DMR; F_1_ hybrid DNA derived from 5 dpp liver. Other details as described in Fig. 2. Data obtained from 7.5 and 14.5 dpc BxC embryos and adult BxC liver and brain are shown in Additional file 8: Figure S2. Reciprocal cross-data obtained from 13.5 dpc CxB embryos are shown in Additional file 10: Figure S4

At *Ndn*, we analyzed the methylation status of 17 CpG dyads located 5′ relative to the start codon; these 17 CpGs had previously been shown to be differentially methylated in multiple adult tissues, including brain, where *Ndn* is expressed, as well as in tissues without *Ndn* expression such as liver and heart [10]. We therefore assessed CpG dyad methylation in tissues derived from embryos, neonatal liver and brain, and adult brain. In every tissue analyzed, the level of methylation on the maternal vs. paternal alleles was significantly different, confirming that this region is differentially methylated throughout development and in multiple tissue types (Fig. 3a). The highest level of methylation we detected was on the maternal allele in 5 dpp liver; methylation of maternal alleles in 5 dpp liver was significantly higher than on maternal alleles derived from 5 dpp or adult brain (Mann–Whitney *U* test, *P* = 0.0108, 0.0271), although this could be attributed to the small sample size. Overall, 39.0% of the CpG dyads at the *Ndn* DMR were hemimethylated on both parental alleles (Additional file 2).

We additionally assessed methylation at 29 CpG dyads located at the 3′ end of the CpG island associated with the maternally methylated *Peg12* gene [30, 32]. Our analysis showed that the maternal allele has significantly more methylation than the paternal allele in embryonic, neonatal and adult tissues (Fig. 3b). In addition, embryonic tissues have significantly less methylation on the maternal allele than neonatal and adult tissues. Overall, 35.4% of the CpG dyads at the *Peg12* DMR were hemimethylated on both parental alleles (Additional file 2). Hemimethylation levels at the maternally methylated *Ndn* and *Peg12* DMRs were not significantly different from each other (*P *= 0.1745), although hemimethylation at both of these maternally methylated secondary DMRs was significantly higher than most of the paternally methylated secondary DMRs we analyzed, with the exception of *Cdkn1c* which contained the highest levels of hemimethylation amongst the loci examined in our study. Combined, these data support the hypothesis that high levels of hemimethylation are characteristic of both maternally and paternally methylated secondary DMRs located throughout the mouse genome and may be a unique epigenetic feature that further distinguishes secondary DMRs from primary DMRs.

### Hemimethylation levels are low at both paternally and maternally methylated primary DMRs associated with imprinted loci

**Fig. 4:**
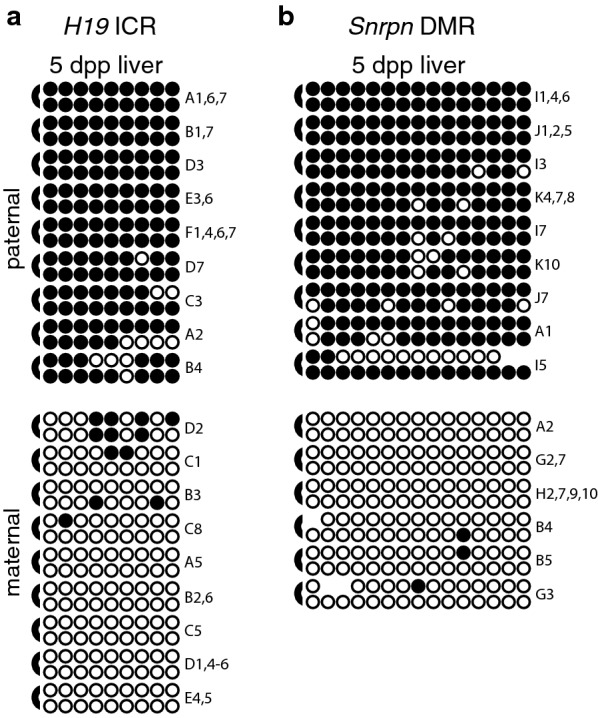
The primary DMRs associated with *H19* and *Snrpn* display low levels of hemimethylation. Details as described in Fig. 2. Data shown are from DNA derived from 5 dpp BxC liver. Data obtained from 7.5 and 14.5 dpc BxC embryos and adult BxC liver are shown in Additional file 9: Figure S3. Reciprocal cross-data obtained from 13.5 dpc CxB embryos are shown in Additional file 10: Figure S4

**Fig. 5:**
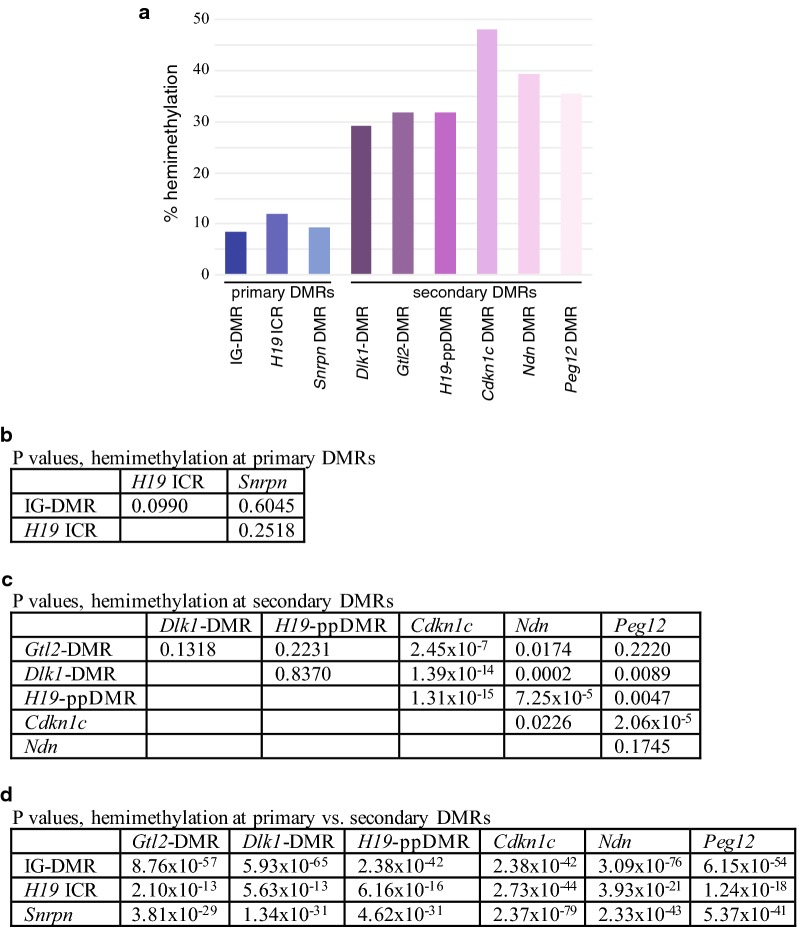
Hemimethylation levels at primary vs. secondary DMRs are significantly different. **a** Hemimethylation levels at primary and secondary DMRs. Chi square tests of independence reveal that hemimethylation levels at primary DMRs are not significantly different from each other (**b**), hemimethylation levels at secondary DMRs show some significant differences (**c**), and that the differences in hemimethylation levels at primary vs. secondary DMRs are highly significant (**d**)

To determine if the high levels of hemimethylation are a unique feature of secondary DMRs associated with imprinted genes, thereby distinguishing them further from primary DMRs, we examined the methylation status of CpG dyads associated with primary DMRs. We assessed DNA methylation at 9 CpG dyads located at the 5′ end of the *H19* imprinting control region (ICR) and 15 CpG dyads located within the *Snrpn* DMR [11, 33]. We observed relatively low levels of hemimethylation at both loci: 11.9% and 9.3% of the CpG dyads were hemimethylated within the paternally methylated *H19* ICR and the maternally methylated *Snrpn* DMR, respectively (Fig. 4; Additional file 2). No significant differences were detected when comparing hemimethylation levels at primary DMRs associated with *Dlk1*/*Gtl2* IG-DMR, *H19* or *Snrpn* (Fig. 5b; [24]). In contrast, the difference in hemimethylation levels at either paternally or maternally methylated primary DMRs when compared to any of the secondary DMRs we analyzed was highly significant (Fig. 5d) and the differences were notably more significant between primary vs. secondary DMRs than between secondary DMRs (Fig. 5c). These results are consistent with the hypothesis that high levels of hemimethylation are characteristic of the variably methylated secondary DMRs but are not associated with primary DMRs. In further support of our theory that high levels of hemimethylation are a unique feature of secondary DMRs associated with imprinted genes, preliminary data show that the level of hemimethylation at tissue-specific DMRs is similar to hemimethylation levels at primary DMRs and is significantly lower than those observed at secondary DMRs (T. Davis, data not shown).

### 5-hydroxymethylcytosine is enriched within paternally methylated secondary DMRs

**Fig. 6:**
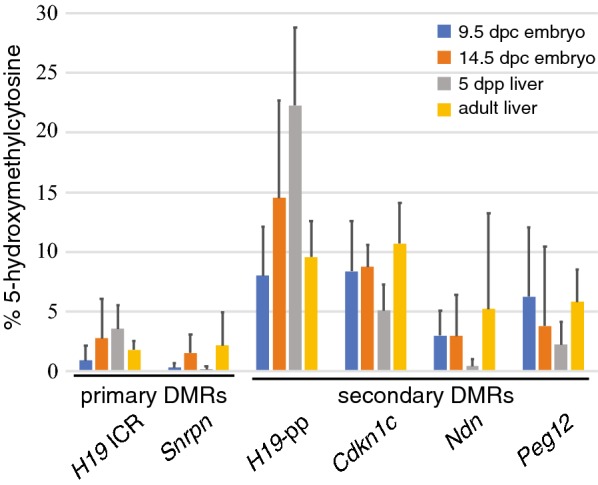
5-hydroxymethylcytosine is enriched at paternally methylated secondary DMRs. Average 5hmC levels and standard deviations for primary DMRs associated with the paternally methylated *H19*-ICR, the maternally methylated *Snrpn* DMR, the paternally methylated *H19*-pp and *Cdkn1c* DMRs and the maternally methylated *Ndn* and *Peg12* DMRs in DNA derived from 9.5 and 14.5 d.p.c. embryos and from 5 d.p.p. and adult liver

We hypothesized that the variability in DNA methylation patterns and the high levels of hemimethylation at secondary DMRs may be the result of 5-hydroxymethylcytosine (5hmC) at these loci, which could result in both passive and active demethylation [25–29]. We tested this hypothesis by assessing the relative levels of 5mC and 5hmC at CpGs located in *Msp*I sites within both primary and secondary DMRs associated with imprinted genes. To conduct this analysis, we glucosylated genomic DNA, digested glucosylated and unglucosylated samples with *Msp*I, *Hpa*II or no enzyme, amplified the resulting products using qPCR and calculated percent 5hmC based on the method previously described by Magalhães et al. [34]. We conducted our analyses across four developmental stages, and the data shown in Fig. 6 represent average 5hmC levels from multiple experiments performed using three independent biological samples at each developmental stage. We found low levels of 5hmC at the primary DMRs associated with *H19* and *Snrpn*, consistent with the low levels we had previously detected at the primary IG-DMR associated with the *Dlk1*/*Gtl2* imprinting cluster (Fig. 6; [24]). Significantly higher levels of 5hmC were detected at the paternally methylated secondary DMRs associated with *H19* and *Cdkn1c* when compared to the two primary DMRs analyzed in this study (Mann–Whitney *U* test, *P *< 0.0001 for all primary DMR vs. secondary DMR combinations). In contrast, both of the maternally methylated secondary DMRs we analyzed, *Ndn* and *Peg12*, displayed intermediate levels of 5hmC. The levels of 5hmC associated with the maternally methylated *Ndn* and *Peg12* secondary DMRs were significantly higher than the levels detected at the maternally methylated *Snrpn* primary DMR (*P *= 0.0067 and 0.0001, respectively), but were not significantly different than the levels detected at the paternally methylated *H19*-ICR (*P *= 0.8650 and 0.0735, respectively). These data suggest that there may be a difference in the degree to which 5hmC is enriched at paternally vs. maternally methylated secondary DMRs.

### Sequence composition analysis does not identify significant differences between paternally vs. maternally methylated secondary DMRs

Our data analyses illustrated that the difference in hemimethylation levels at primary DMRs vs. secondary DMRs is highly significant. In contrast, hemimethylation levels are not significantly different between the primary DMRs analyzed in this study. Although there are neither significant differences in hemimethylation levels at the two maternally methylated secondary DMRs nor at the paternally methylated secondary DMRs associated with *Dlk1*, *Gtl2* and *H19*, there are significant differences in hemimethylation levels when comparing the paternally vs. maternally methylated secondary DMRs. Given this distinction, we undertook an analysis of sequence composition to determine if variation in dinucleotide composition correlated with differences in hemimethylation levels.

For our dataset, we utilized sequences defined by Xie et al. [35] which were identified in their genome-wide allele-specific methylation study and correlated in size with differentially methylated regions associated with imprinted loci in other studies. Our dataset included 3 paternally methylated and 16 maternally methylated primary DMRs as well as 7 paternally methylated and 9 maternally methylated secondary DMRs (Additional file 3). For the sequence composition analysis, we took the same approach as Kobayashi et al. [36], who investigated sequence composition between paternally vs. maternally methylated primary DMRs. They noted a significantly higher frequency of CpG dinucleotides within maternally methylated primary DMRs as compared to paternally methylated primary DMRs (*P *= 0.0300; [36]). In contrast, we did not identify any significant differences in CpG frequency when comparing paternal vs. maternal secondary DMRs or any combination of primary vs. secondary DMR (Table 1). Overall, these results led us to conclude that secondary DMRs do not have significantly fewer CpG dinucleotides than primary DMRs, therefore, CpG content is unlikely to be a primary factor regulating methylation stability at these loci. Furthermore, we did not find any significant differences in sequence composition between paternally vs. maternally methylated secondary DMRs, therefore, sequence composition is unlikely to account for the variation we observed in hemimethylation frequency between secondary DMRs. In addition to the significant differences in sequence composition between paternally and maternally methylated primary DMRs originally noted by Kobayashi et al. [36], we also detected additional significant differences in dinucleotide content between paternally methylated primary DMRs and paternally or maternally methylated secondary DMRs (Table 1). Notably, the small sample size of 3 paternally methylated DMRs may have affected the results, as all the significant differences we identified were between the paternally methylated DMRs and other DMR categories; further analysis would be necessary to determine if these non-CpG differences are biologically relevant.

**Table 1:** Comparison of dinucleotide content within primary vs. secondary DMRs (*P* values)

	CpG	GpC	ApT	TpA	ApA+ TpT	ApC+ GpT	ApG+ CpT	CpA+ TpG	CpC+ GpG	GpA+ TpC
Pat1° vs. Mat1°	0.0240	0.0139	0.0004	0.0444	0.4542	0.2515	0.9681	0.0074	0.0461	0.7065
Pat2° vs. Mat2°	0.4963	0.9369	0.9178	0.9987	0.3756	0.1281	0.2590	0.9287	0.7013	0.6743
Pat1° vs. Pat2°	0.0861	0.0835	0.0102	0.0387	0.2490	0.3482	0.6532	0.0768	0.0486	0.5086
Mat1° vs. Mat2°	0.7422	0.6371	0.7383	0.4821	0.6925	0.1175	0.2031	0.8800	0.9646	0.9638
Pat1° vs. Mat2°	0.1057	0.0472	0.0160	0.0218	0.7908	0.0570	0.2808	0.0187	0.1886	0.7569
Pat2° vs. Mat1°	0.5735	0.7343	0.8067	0.5679	0.4508	0.6926	0.6236	0.9804	0.6192	0.6434

(*Pat1*° paternally methylated primary DMR, *Mat1°* maternally methylated primary DMR, *Pat2°* paternally methylated secondary DMR, *Mat2°* maternally methylated secondary DMR. Frequency of each dinucleotide was determined as described in “Methods”. *P* values were calculated using a two-tailed *t* test for independent samples as described in “Methods”. Raw data are found in Additional file 3

## Discussion

The research described herein focuses on characterizing DNA methylation at secondary DMRs associated with imprinted genes. Secondary DMRs, which acquire their differentially methylated status post-fertilization, display significantly more variation in their methylation patterns than do primary DMRs [10–13, 24, 37]. Despite the variability in methylation at these loci, they appear to play a critical role in maintaining imprinted expression at the individual genes with which they are associated as loss of imprinting at these genes can result either from deletion of the secondary DMR or from its loss of methylation due to mutations in *Dnmt1* or deletion of the corresponding primary DMR [18, 37–41]. Understanding how methylation at secondary DMRs influences imprinted gene expression despite the absence of highly stable methylation patterns at these sites is, therefore, important. Our investigation into the variable nature of DNA methylation at imprinted loci has identified high levels of hemimethylation specifically at paternally and maternally methylated secondary DMRs, which we hypothesize is connected to the presence of 5-hydroxymethylcytosine leading to demethylation and hence the observed methylation asymmetries. In this study, we obtained data consistent with this hypothesis: all three of the primary DMRs analyzed in this study and our previous work had low levels of 5hmC, while more 5hmC was prevalent at the paternally methylated secondary DMRs associated with *H19*, *Cdkn1c* and *Dlk1* ([24] and data herein). However, we did not detect similarly high levels of 5hmC at the *Gtl2* secondary DMR, nor at the maternally methylated *Ndn* or *Peg12* secondary DMRs. These data may highlight a difference in the methylation state at paternally vs. maternally methylated secondary DMRs. It is also possible that these data are not representative of the overall level or distribution of 5hmC across these loci, as the scope of our 5hmC analysis was limited to CpGs located within *Msp*I restriction sites. In addition, our assay was not allele-specific, preventing us from assessing the distribution of 5hmC on the methylated vs. unmethylated allele. To resolve these questions, we are currently undertaking an oxidative bisulfite sequencing approach to interrogate these loci more broadly and determine if 5hmC is enriched at secondary DMRs and what its distribution is on the parental alleles to better determine if the presence of 5hmC could be driving passive and/or active demethylation at secondary DMRs. In support of this hypothesis, we detected a significant enrichment of 5hmC on both parental alleles at two secondary DMRs but not at the primary DMR examined in our pilot study (Raymond and Davis, unpublished data).

Regardless of the causative mechanism, the high incidence of hemimethylation at secondary DMRs indicates that methylation is not well maintained at these loci. Previous research has shown that UHRF1 binds with high affinity to hemimethylated CpGs, recruiting DNMT1 specifically during S-phase and ensuring the maintenance of methylation [42–44]. The activity of UHRF1, therefore, helps to promote epigenetic stability which is necessary for the maintenance of imprinting marks. However, because recruitment of DNMT1 to hemimethylated DNA is S-phase dependent, UHRF1 would not be able to stimulate maintenance methylation when loss of methylation occurs outside of DNA replication and would not be able to correct for the loss of DNA methylation in post-replicative DNA. Furthermore, although UHRF1 has been shown to bind 5hmC just as well as 5mC in vitro [45], UHRF2 has higher affinity for 5hmC and does not recruit DNMT1 to replication foci [44]. Therefore, it is possible that UHRF2 competes with UHRF1 at secondary DMRs containing 5hmC, preventing maintenance methylation at these loci and contributing to high frequency of hemimethylation at these loci. Additionally, the E3 ligase activity of UHRF2 is activated by its association with hemimethylated 5hmC [46], which may lead to increased activity of TET2 resulting in successive oxidation and eventual loss of methylation following base excision repair [27, 47]. Enrichment of 5hmC at secondary DMRs could, therefore, contribute to the active demethylation of these loci via its association with UHRF2.

Given all the possible factors that could contribute to a loss of methylation in the presence of 5hmC, this raises the question as to how methylation is maintained in the absence of symmetrical DNA methylation patterns since epigenetic stability is dependent on consistent propagation of DNA methylation profiles. Indeed, it has been shown that differentiated cells display a strong preference for concordant methylation [48]. Therefore, there must be a mechanism for maintaining methylation at CpG dyads within secondary DMRs despite the high level of hemimethylation, as the overall level of DNA methylation at these loci is consistent throughout development once it is established ([12, 13, 16, 24] and data herein). We suggest that primary DMRs, which are responsible for the parent of origin-specific acquisition and/or maintenance of DNA methylation at secondary DMRs during post-implantation [12, 49–51], must also act throughout development to consistently drive the remethylation of these sequences, countering the effects of demethylation and thereby maintaining the differentially methylated state at these loci. This hypothesis could be tested by conditionally knocking out a primary DMR after methylation is acquired at its corresponding secondary DMR(s) to determine if methylation levels continue to be maintained.

While Dnmt1 is responsible for methylation maintenance, mutations in *Dnmt1* have uncoupled its ability to function in maintaining global DNA methylation vs. methylation at gametic DMRs, specifically those associated with imprinted genes [52, 53]. For example, the *Dnmt1*^*P*^ allele has the ability to maintain methylation at gametic DMRs despite having greatly reduced levels of global DNA methylation, illustrating that the mouse-specific motif LESHTV within the intrinsically disordered domain is required for maintaining global DNA methylation [53]. These results suggest that Dnmt1 may be functioning differently at different genomic sequences. Indeed, Dnmt1 has been shown to interact with a large number of other proteins and its ability to function at global genomic loci vs. gametic DMRs may be influenced by its ability to interact with different partners via its intrinsically disordered domain [54]. This hypothesis is supported by the observation that the amino acid substitutions associated with the P allele result in a local increase in disorder [54], potentially affecting the proteins with which Dnmt1 can interact and therefore compromising its function globally without affecting its ability to act at gametic DMRs. Furthermore, the evidence that the maintenance of some DNA methylation may require both Dnmt1 and Dnmt3 [55, 56] is consistent with our hypothesis that secondary DMRs require remethylation throughout development.

## Conclusions

Our analyses illustrate that the variable DNA methylation patterns observed at secondary DMRs associated with imprinted genes are a result of high levels of hemimethylation which we show is a generalizable characteristic of both paternally and maternally methylated secondary DMRs. Hemimethylation could result from active demethylation and/or from a failure of maintenance methylation mechanisms and should, in theory, lead to loss of methylation over time. However, despite the high levels of hemimethylation we observed at secondary DMRs, overall methylation levels do not change significantly throughout development once methylation is acquired. We therefore conclude that parent of origin-specific methylation at secondary DMRs must be reacquired to counteract the mechanisms leading to hemimethylation at these loci, highlighting the complexities of DNA methylation dynamics at imprinted genes. Further research is necessary to identify the components of the DNA methylation machinery that play a role in methylation acquisition and maintenance at secondary DMRs as well as other factors involved.

## Methods

### Mice

C57BL/6J (B) and *Mus musculus castaneus* (C) mice were purchased from the Jackson Laboratory. Natural matings between C57BL/6J and *Mus musculus castaneus* were used to generate BxC or CxB F_1_ hybrid tissues used for bisulfite analyses. For all F_1_ hybrid samples, the maternal allele is located on the left. Ethical approval for procedures involving animals was granted by the Bryn Mawr College Institutional Animal Care and Use Committee, PHS Welfare Assurance Number A3920-01.

### DNA purification, template preparation and bisulfite analysis

DNA was isolated from 7.5 dpc embryos using the DNeasy Blood & Tissue Kit (Qiagen. Germantown, MD, cat#69504). DNA was isolated from 9.5, 13.5 and 14.5 dpc embryos and from 5 dpp and adult liver and brain following proteinase K digestion and a series of phenol/chloroform extractions as described previously [57]. Prior to bisulfite mutagenesis, complementary strands of DNA were covalently attached as described by Laird et al. [58]; specific restriction enzymes and oligonucleotide sequences are listed in Additional file 4. For each sequence to be analyzed, 0.5 µg of genomic DNA was digested with the specified restriction enzyme and ligated to 1 µg of the appropriate phosphorylated hairpin linker. 0.5 µg of hairpin linked, ligated DNA was denatured by incubating in freshly prepared 3 M NaOH for 20 min at 42 °C, then subjected to bisulfite mutagenesis using an EZ DNA Methylation-Direct kit (Zymo Research, Irvine, CA, cat#D5020). All mutagenized DNAs were subjected to multiple independent PCR amplifications to ensure analysis of different strands of DNA, as subclones obtained from the same PCR reaction and displaying the same sequence, including the same methylation pattern, cannot be definitively proven to derive from different template; subclones derived from independent PCR amplifications are distinguished by different letters of the alphabet. For *Peg12*, the hairpin linker included a random barcode that allowed for the identification of redundant sequences [59]. Data from multiple independent tissue samples derived from the same developmental stage were combined, as we did not detect variation between biological replicates when comparing methylation and hemimethylation frequencies. Primer pairs used for nested amplification of mutagenized DNA were designed to incorporate at least one SNP as well as CpG dinucleotides within the previously analyzed DMRs [10–12, 32, 33]. Genomic coordinates, primers, PCR cycling conditions and expected second round PCR product size for each DMR are detailed in Additional file 5. Subcloning of amplified products was achieved using a pGEM-T Easy vector (Promega Corporation, Madison, WI, cat#A1360). Sequencing reactions were conducted by Genewiz (South Plainfield, NJ) or using a Thermo Sequenase Cycle Sequencing Kit (Affymetrix, Cleveland, OH, cat#78500) and analyzed on a 4300 DNA Analyzer (LI-COR Biosciences, Lincoln, NE). Sequence polymorphisms used to distinguish C57BL/6J vs. *Mus musculus castaneus* DNA (B/C): *H19*-ppDMR, T/C at chr7: 142,578,903; *H19* ICR, A/G at chr7:142,581,765, G/A at chr7:142,581,852; *Cdkn1c*, T/G at chr7:143,461,451; *Ndn*, C/G at chr7:62,348,216, A/G at 62,348,271; *Peg12*, T/C at chr7:62,463,607; *Snrpn*, G/T at chr7:60,005,215, C/T at chr7:60,005,265, C/T at chr7:60,005,282. Bisulfite conversion efficiency was determined for each locus analyzed; in total, 662 cytosines were detected at 74,735 non-CpG cytosine locations for a failed conversion rate of 0.89%, similar to error rates reported previously [24, 59]. Percent methylation was calculated based on data obtained from both complementary strands. Percent hemimethylation was calculated by dividing the number of hemimethylated CpG dinucleotides by the number of hemimethylated + homomethylated CpG dyads. Percent methylation for each strand was calculated and the raw data from each parental allele at each developmental stage was ranked and assessed for statistically significant differences using a Mann–Whitney *U* test (http://vassarstats.net/utest.html). Chi square tests of independence were conducted in Microsoft Excel, utilizing the raw number of homo- and hemimethylated CpG dyads at different loci.

### 5-hydroxymethylation analysis

For 5-hydroxymethylation analyses, DNA was isolated from 9.5 dpc embryos, 14.5 dpc embryos, 5 dpp liver and adult liver as described above. DNA derived from three different genetic backgrounds [C57BL/6J, B6x(CAST or CAST12) and (CAST or CAST12)xB] was used as the three biological replicates. 5-hydroxymethylation levels were assessed using an EpiMark 5hmC and 5mC Analysis Kit (NEB, Ipswich, MA, cat#E3317). Briefly, 2.5 µg genomic DNA was glucosylated using 30 units of T4 ß-glucosyltransferase at 37 °C overnight. Glucosylated and unglucosylated control DNA was treated with *Msp*I, *Hpa*II or no restriction endonuclease at 37 °C overnight. Following treatment with proteinase K, products were amplified via quantitative PCR (StepOnePlus, Applied Biosystems). Primers and PCR cycling conditions used are detailed in Additional file 6. qPCR was performed in triplicate for each of the three independent biological samples. Amount of 5mC and 5hmC was calculated according to Magalhães et al. [34]. 5hmC levels from each locus were calculated and pairwise combinations of 5hmC levels were ranked and assessed for statistically significant differences between loci using a Mann–Whitney *U* test (http://vassarstats.net/utest.html). Genomic coordinates for *Msp*I/*Hpa*II sites: *H19*-ppDMR, chr7:142,578,770; *H19* ICR, chr7:142,581,144; *Cdkn1c* DMR, chr7:143,461,739; *Ndn* DMR, chr7:62,348,492; *Peg12* DMR, chr7:62,463,521; *Snrpn* DMR, chr7:60,005,094.

### Sequence composition analysis

Sequences were obtained from GRCm38/mm10 based on data coordinates provided in Xie et al. [35]. Dinucleotide sequence composition was obtained using the Genomatix Software Suite (http://www.genomatix.de/cgi-bin/tools/tools.pl). Raw data (Additional file 3) were analyzed using a two-tailed *t* test for independent samples to identify significant differences between samples (http://vassarstats.net/tu.html).

## Supplementary information

**Additional file 1.** Methylation levels at imprinted DMRs generally do not vary across development. Mann–Whitney *U* tests were performed to compare methylation patterns on maternal and paternal alleles at each primary and secondary DMR across development. In general, methylation levels on each parental allele did not significantly change across development, illustrating that overall levels of methylation are maintained developmentally once they are established.

**Additional file 2.** Hemimethylation data from each locus across development. Individual pages contain data for number of hemimethylated sites at the *H19*-ppDMR, *Cdkn1c* DMR, *Ndn* DMR, *Peg12* DMR, *H19* ICR and *Snrpn* DMR. Hemimethylation frequencies were determined from raw data illustrated in Figs. 2, 3, 4 and Additional file 7: Figure S1; Additional file 8: Figure S2; Additional file 9: Figure S3; Additional file 10: Figure S4. Frequencies were calculated for maternal and paternal alleles at each developmental stage and combined to obtain total hemimethylation values.

**Additional file 3.** Dinucleotide frequencies at paternally- and maternally-methylated DMRs associated with imprinted genes. Frequencies were calculated as described in “Methods” using data coordinates provided in Xie et al. [35].

**Additional file 4.** Restriction enzymes and hairpin linker sequences for covalent attachment of complementary DNA strands for each DMR analyzed in this study.

**Additional file 5.** Primer and PCR cycling conditions for amplification of bisulfite-mutagenized DNA for each DMR analyzed in this study and relative positions of amplicons to CpG islands/DMRs and transcription units.

**Additional file 6.** Primers and PCR cycling conditions for 5hmC analyses for each DMR analyzed in this study.

**Additional file 7: Figure S1.** The paternally methylated secondary DMRs associated with *H19* and *Cdkn1c* display a high level of hemimethylation. Bisulfite mutagenesis and sequencing of F_1_ hybrid DNA derived from 14.5 dpc BxC embryos and adult BxC liver. Individual circles in each row represent one of the potentially methylated CpG dinucleotides analyzed at the *H19*-ppDMR (A) or the *Cdkn1c* DMR (B), and each paired row of circles represents the complementary strands of an individual subclone; semi-circles to the right or left indicate the location of the linker connecting the complementary strands. Filled circles represent methylated cytosines, open circles represent unmethylated cytosines, absent circles represent ambiguous data. Alphanumeric labels identify subclones analyzed; letters represent independent amplification reactions, while numbers represent individual subclones. Subclones derived from the same amplification that have identical sequence and methylation patterns are grouped together, as it was not possible to determine if these amplicons were derived from the same or different template molecules.

**Additional file 8: Figure S2.** The maternally methylated secondary DMRs associated with *Ndn* and *Peg12* display a high level of hemimethylation. (A) Methylation status at the *Ndn* DMR; F_1_ hybrid DNA derived from 14.5 dpc BxC embryos and adult brain. (B) Methylation status at the *Peg12* DMR; F_1_ hybrid DNA derived from 7.5 and 14.5 dpc BxC embryos and adult BxC liver. Other details as described in Additional file 7: Figure S1.

**Additional file 9: Figure S3.** The primary DMRs associated with *H19* and *Snrpn* display low levels of hemimethylation. Data shown are from DNA derived from 7.5 and 14.5 dpc BxC embryos and adult liver. Details as described in Additional file 7: Figure S1.

**Additional file 10: Figure S4.** Reciprocal crosses illustrate that allele-specific methylation is dependent on parental origin, not strain. Data shown are from DNA derived from a 13.5 dpc CxB embryo. (A) Paternally and maternally methylated secondary DMRs. (B) Paternally and maternally methylated primary DMRs. Details as described in Additional file 7: Figure S1.

## Data Availability

All data generated or analyzed during this study are included in this published article and its Additional files.

## Abbreviations

ICR: imprinting control region
DMR: differentially methylated region
IG-DMR: intergenic DMR
dpc: days post-coitum
dpp: days post-partum
B: C57BL/6J
C: *Mus musculus castaneus*
PCR: polymerase chain reaction
5mC: 5-methylcytosine
5hmC: 5-hydroxymethylcytosine
