# Supplementary material for: Hemimethylation of CpG dyads is characteristic of secondary DMRs associated with imprinted loci and correlates with 5-hydroxymethylcytosine at paternally methylated sequences
Source: Epigenetics Chromatin. 2019 Oct 17;12:64. doi: 10.1186/s13072-019-0309-2 (PMC6796366; doi:10.1186/s13072-019-0309-2)
Supplement: Supplementary file 4 — Additional file 4. Restriction enzymes and hairpin linker sequences for covalent attachment of complementary DNA strands for each DMR analyzed in this study. [file 13072_2019_309_MOESM4_ESM.docx]

**Additional File 3.** Restriction enzymes and hairpin linker sequences for covalent attachment of complementary DNA strands.

| locus analyzed | restriction enzyme^a^,  digestion & heat inactivation temperatures | hairpin linker |
| --- | --- | --- |
| *H19*-ppDMR | *Ban*I (#R0118S)  37°C, 65°C | 5’-GCACAGCGATGCgttcgaGCATCGCT-3’ |
| *Cdkn1c* DMR | *Sac*I-HF (#R3156S)  37°C, 65°C | 5’- AGCGATGCgttcgaGCATCGCTAGCT -3’ |
| *Ndn* DMR | *Bpu*10I (#R0649S)  37°C, 80°C | 5’- TGAAGCGATGCgttcgaGCATCGCT -3’ |
| *Peg12* DMR | *Bsa*WI (#R0567S)  60°C, 80°C | 5’- CCGGAGCGATGCDDDDDDDGCATCGCT -3’ |
| *H19* ICR | *Ban*I (#R0118S)  37°C, 65°C | 5’- GTACAGCGATGCgttcgaGCATCGCT -3’ |
| *Snrpn* DMR | *Apo*I (#R0566S)  50°C, 80°C | 5’- AATTAGCGATGCgttcgaGCATCGCT -3’ |

For each DMR analyzed, genomic DNA was digested with the noted restriction enzyme and the digested products were ligated to staggered ends present in the hairpin linker to achieve covalent attachment of the complementary strands of DNA in order to obtain methylation data for each CpG dyad.

^a^Restriction enzymes were purchased from NEB, Ipswitch, MA.
