# Supplementary material for: Hemimethylation of CpG dyads is characteristic of secondary DMRs associated with imprinted loci and correlates with 5-hydroxymethylcytosine at paternally methylated sequences
Source: Epigenetics Chromatin. 2019 Oct 17;12:64. doi: 10.1186/s13072-019-0309-2 (PMC6796366; doi:10.1186/s13072-019-0309-2)
Supplement: Supplementary file 5 — Additional file 5. Primer and PCR cycling conditions for amplification of bisulfite-mutagenized DNA for each DMR analyzed in this study and relative positions of amplicons to CpG islands/DMRs and transcription units. [file 13072_2019_309_MOESM5_ESM.docx]

**Additional File 4.** Primer and PCR cycling conditions for amplification of bisulfite-mutagenized DNA and relative positions of amplicons to CpG islands/DMRs and transcription units.

| locus analyzed | round | primers | cycling conditions |
| --- | --- | --- | --- |
| *H19*-ppDMR  CpG #2-9^a^  (Tremblay 1997)  chr7:142,578,866-142,578,672^b^  final product:  672 bp | 1^st^ | 5’-GGTTAATTTTTAGTTTTTGTTTTA-3’  5’-CCCAACCTTTATCCTAATCTAC-3’ | 94°C 2 min  55°C 1 min  72°C 1 min  repeat 2x  94°C 30 sec  55°C 1 min  72°C 1 min  repeat 30x  72°C 10 min |
|  | 2^nd^ | 5’-GTTTTAATAAGGTAGTAGTTAATTTG-3’  5’-CTACTTAACTAACCTTATCTAACC-3’ | 94°C 30 sec  50°C 1 min  72°C 1 min  repeat 35x  72°C 10 min |
| *Cdkn1c* DMR  CpG #1-29^a^  (Bhogal 2004)  chr7:143,461,477-143,461,288^b^  final product:  594 bp | 1^st^ | 5’-GAAGGTAGATATTTAGGTTTGGGTG-3’  5’-CCTAAATATAAAAAATAAATCCAACCAC-3’ | 94°C 2 min  55°C 1 min  72°C 1 min  repeat 2x  94°C 30 sec  55°C 1 min  72°C 1 min  repeat 30x  72°C 10 min |
|  | 2^nd^ | 5’-GGTTTGGGTGTAGAGGGTGG-3’  5’-CTACCAAAACCAAAACCCAAC-3’ | 94°C 30 sec  60°C 1 min  72°C 1 min  repeat 30x  72°C 10 min |
| *Ndn* DMR  CpG #1-17^a^  (Hanel & Wevrick 2001)  chr7:62,348,214-62,348,412^b^  final product:  561 bp | 1^st^ | 5’-GTTTTGTGTTATATAGGAGATTAGG-3’  5’-CTTTTACATAAACCTAATAATACCCTC-3’ | 94°C 2 min  50°C 1 min  72°C 1 min  repeat 2x  94°C 30 sec  50°C 1 min  72°C 1 min  repeat 30x  72°C 10 min |
|  | 2^nd^ | 5’-GATTAGGAAATTTTTTATATAAGTTTAG-3’  5’-CTTAAACCCCAATAATTAAACTTTAC-3’ | 94°C 30 sec  60°C 1 min  72°C 1 min  repeat 30x  72°C 10 min |
| *Peg12* DMR  3’ end of CGI  (Chai 2001; Brant 2014)^c^  chr7:62,463,844-62,463,586^b^  final product:  649 bp | 1^st^ | 5’-TTGTTATAGGTTGGTGTTGTGG-3’  5’-CCTACTACAAATTAATACTATAACC-3’ | 94°C 2 min  50°C 1 min  72°C 1.5 min  repeat 2x  94°C 30 sec  50°C 1 min  72°C 1.5 min  repeat 30x  72°C 10 min |
|  | 2^nd^ | 5’-GGTTATTAGTTGTTAGGGATAAG-3’  5’-CCTAATTCTAAACTATACCCTCC-3’ | 94°C 30 sec  50°C 1 min  72°C 1.5 min  repeat 30x  72°C 10 min |
| *H19* ICR  CpG #7-15^a^  (Tremblay 1997)  chr7:142,581,880-142,581,761^b^  final product:  402 bp | 1^st^ | 5’-gatggttttagaattttataagttag-3’  5’-CCAAAACCCTATAAATCAAATACC-3’ | 94°C 2 min  52°C 1 min  72°C 1 min  repeat 2x  94°C 30 sec  52°C 1 min  72°C 1 min  repeat 30x  72°C 10 min |
|  | 2^nd^ | 5’-GGTTTTATGAAGTTTATGATTATGG-3’  5’-CCCATAACTATAAAATCATAAATAATA -3’ | 94°C 30 sec  52°C 1 min  72°C 1 min  repeat 30x  72°C 10 min |
| *Snrpn* DMR  CpG #2-16^a^  (Lucifero 2002)  chr7:60,005,284-60,005,043^b^  final product:  577 bp | 1^st^ | 5’-GAATGTTTTGGTTAAATAGGATGT-3’  5’-TATTATTAGAATTTATAAGTTTAGTTG-3’ | 94°C 2 min  50°C 1 min  72°C 1.5 min  repeat 2x  94°C 30 sec  50°C 1 min  72°C 1.5 min  repeat 30x  72°C 10 min |
|  | 2^nd^ | 5’-AGTATTTAGATTATTTTTTATATTTTTATA-3’  5’-CACAAACCCAACTAACCTTCC-3’ | 94°C 30 sec  50°C 1 min  72°C 1.5 min  repeat 30x  72°C 10 min |

^a^CpG numbers in relationship to previously published data.

^b^Genomic coordinates for the first and last CpG analyzed (GRCm38/mm10).

^c^A differentially methylated *Eag*I site (Chai *et al.*, 2001) is located 30 bp 5’ relative to the first CpG analyzed in this study. CpGs 1-7, 21-24 & 26-29 correspond to CpGs 58-64, 65-68 & 69-72 from Brant *et al*., 2014, and are located at the 3’ end of the CpG island.


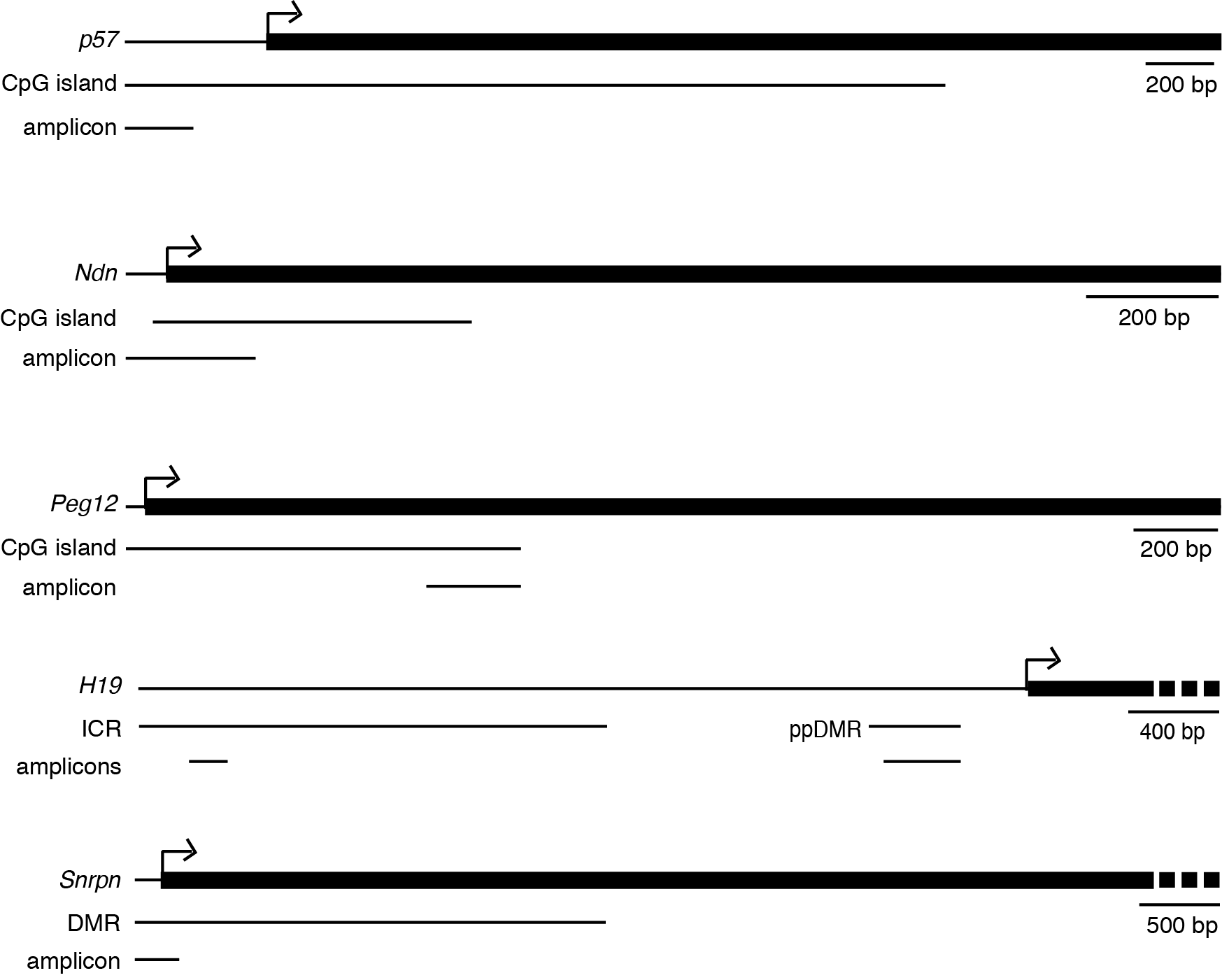


For each imprinted gene, arrow = transcriptional start site, rectangle = transcription unit. Relative positions of CpG islands or non-CpG island DMRs and amplicon analyzed in this study shown as lines below the genetic locus.
