# Supplementary material for: Hemimethylation of CpG dyads is characteristic of secondary DMRs associated with imprinted loci and correlates with 5-hydroxymethylcytosine at paternally methylated sequences
Source: Epigenetics Chromatin. 2019 Oct 17;12:64. doi: 10.1186/s13072-019-0309-2 (PMC6796366; doi:10.1186/s13072-019-0309-2)
Supplement: Supplementary file 6 — Additional file 6. Primers and PCR cycling conditions for 5hmC analyses for each DMR analyzed in this study. [file 13072_2019_309_MOESM6_ESM.docx]

**Additional File 5.** Primers and PCR cycling conditions for 5-hmC analyses.

| region | primers | PCR cycling conditions | PCR product  size |
| --- | --- | --- | --- |
| *H19* ICR | F: 5’- AGGACACCTATGCCCTT -3’  R: 5’- CGCAGCAATTTGGTCTTTC -3’ | 94°C, 30 sec  60°C, 1 min  72°C, 1 min  repeat 30x  72°C, 10 min | 119 bp |
| *Snrpn* DMR | F: 5’- CCATTGCGGCAAGACTA -3’  R: 5’- GGATGCACTTTCACTACTAGAAT -3’ |  | 135 bp |
| *H19*-ppDMR | F: 5’- AGTAGTACTTCAGTAGGATAGGG -3’  R: 5’- AGTTATCTTACAGTCTGGTCTTG -3’ |  | 80 bp |
| *Cdkn1c* DMR | F: 5’- AATATGGCCTGACCCAAAC -3’  R: 5’- AGATCTGTAGCCTGGTCTATAA -3’ |  | 126 bp |
| *Ndn* DMR | F: 5’- GACTGTGAGATGCAGGAC -3’  R: 5’- CTGTTGGGCTGCCATAG -3’ |  | 117 bp |
| *Peg12* DMR | F: 5’- GGGCACAGCTCAGAACTA -3’  R: 5’- CTGGGTGAATCCCTTGGT -3’ |  | 102 bp |

PCR primers were designed to flank individual *Msp*I restriction enzyme recognition sites located within each DMR. Amplification of a unique, appropriately sized product was confirmed on a 10% polyacrylamide gel prior to qPCR experiments. 5-hmC analyses were conducted as described in the Materials and Methods.
