# Supplementary figures and images for: Hemimethylation of CpG dyads is characteristic of secondary DMRs associated with imprinted loci and correlates with 5-hydroxymethylcytosine at paternally methylated sequences
Source: Epigenetics Chromatin. 2019 Oct 17;12:64. doi: 10.1186/s13072-019-0309-2 (PMC6796366; doi:10.1186/s13072-019-0309-2)

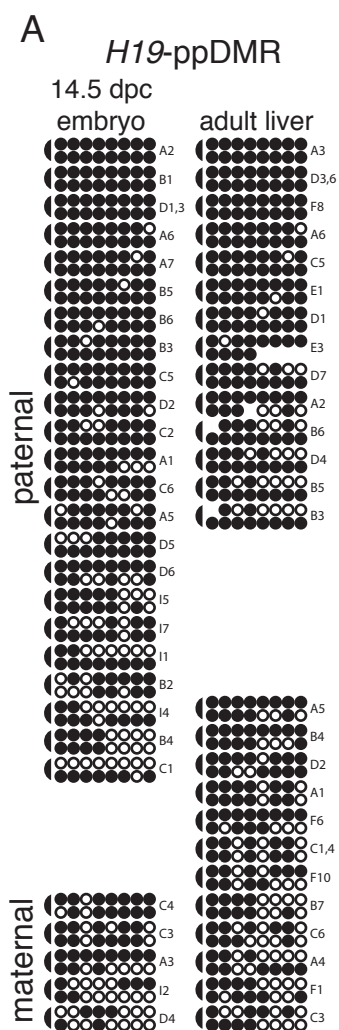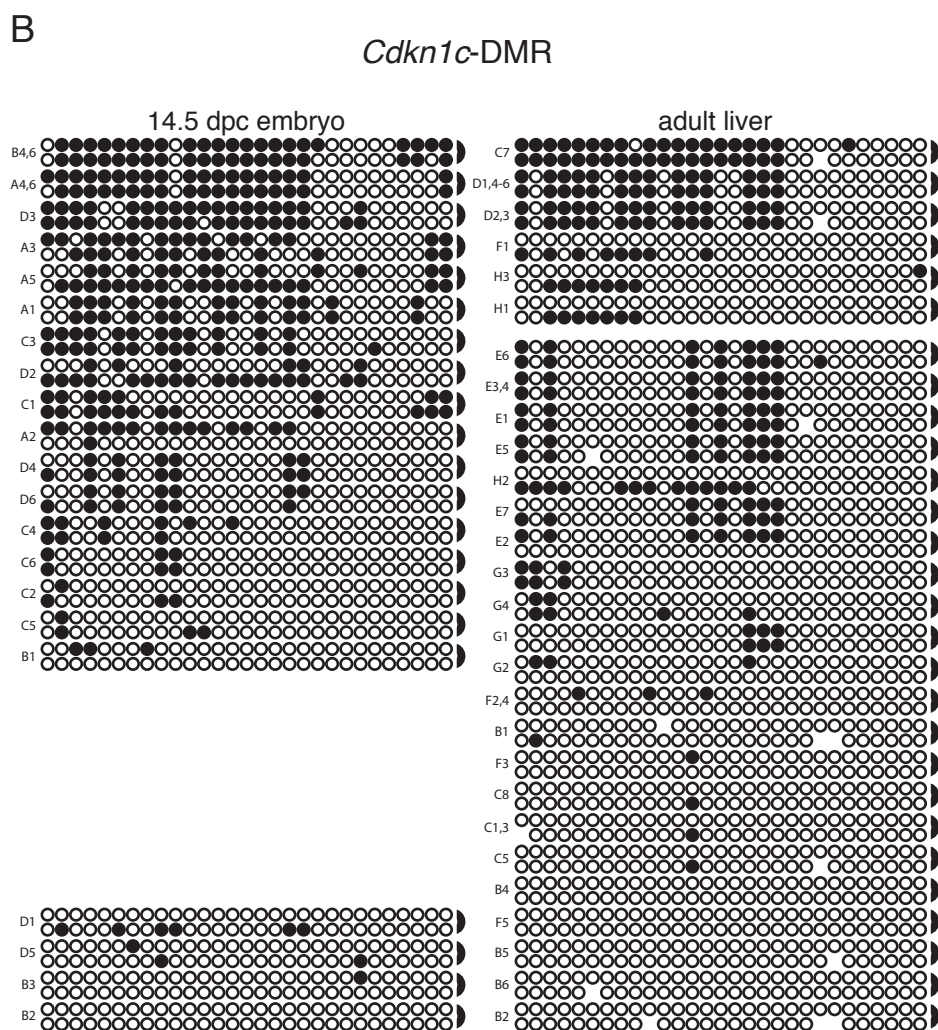

Supplement: Supplementary file 7 — Additional file 7: Figure S1. The paternally methylated secondary DMRs associated with H19 and Cdkn1c display a high level of hemimethylation. Bisulfite mutagenesis and sequencing of F1 hybrid DNA derived from 14.5 dpc BxC embryos and adult BxC liver. Individual circles in each row represent one of the potentially methylated CpG dinucleotides analyzed at the H19-ppDMR (A) or the Cdkn1c DMR (B), and each paired row of circles represents the complementary strands of an individual subclone; semi-circles to the right or left indicate the location of the linker connecting the complementary strands. Filled circles represent methylated cytosines, open circles represent unmethylated cytosines, absent circles represent ambiguous data. Alphanumeric labels identify subclones analyzed; letters represent independent amplification reactions, while numbers represent individual subclones. Subclones derived from the same amplification that have identical sequence and methylation patterns are grouped together, as it was not possible to determine if these amplicons were derived from the same or different template molecules. [file 13072_2019_309_MOESM7_ESM.pdf]

A

*Ndn* DMR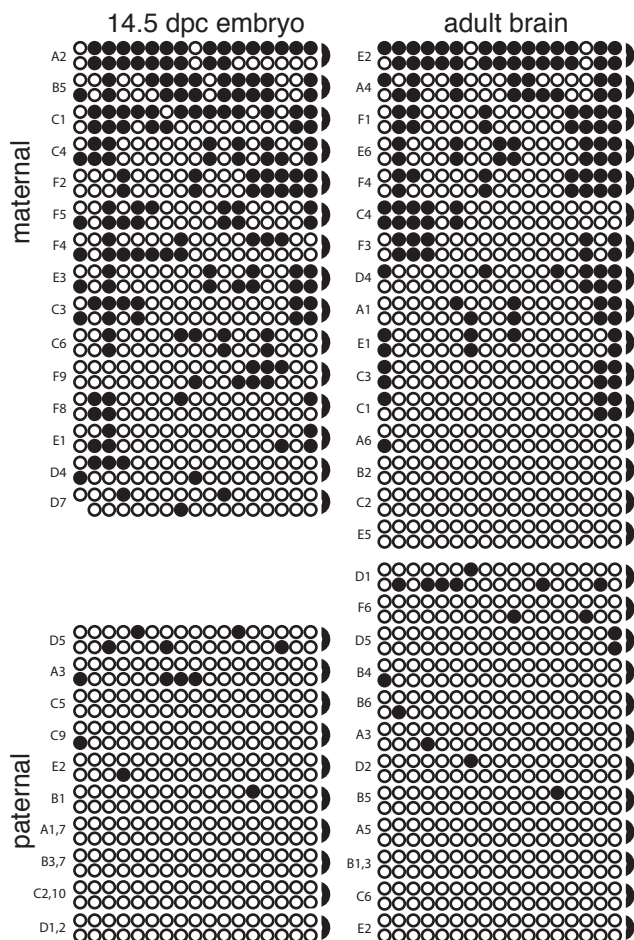

B

*Peg12* DMR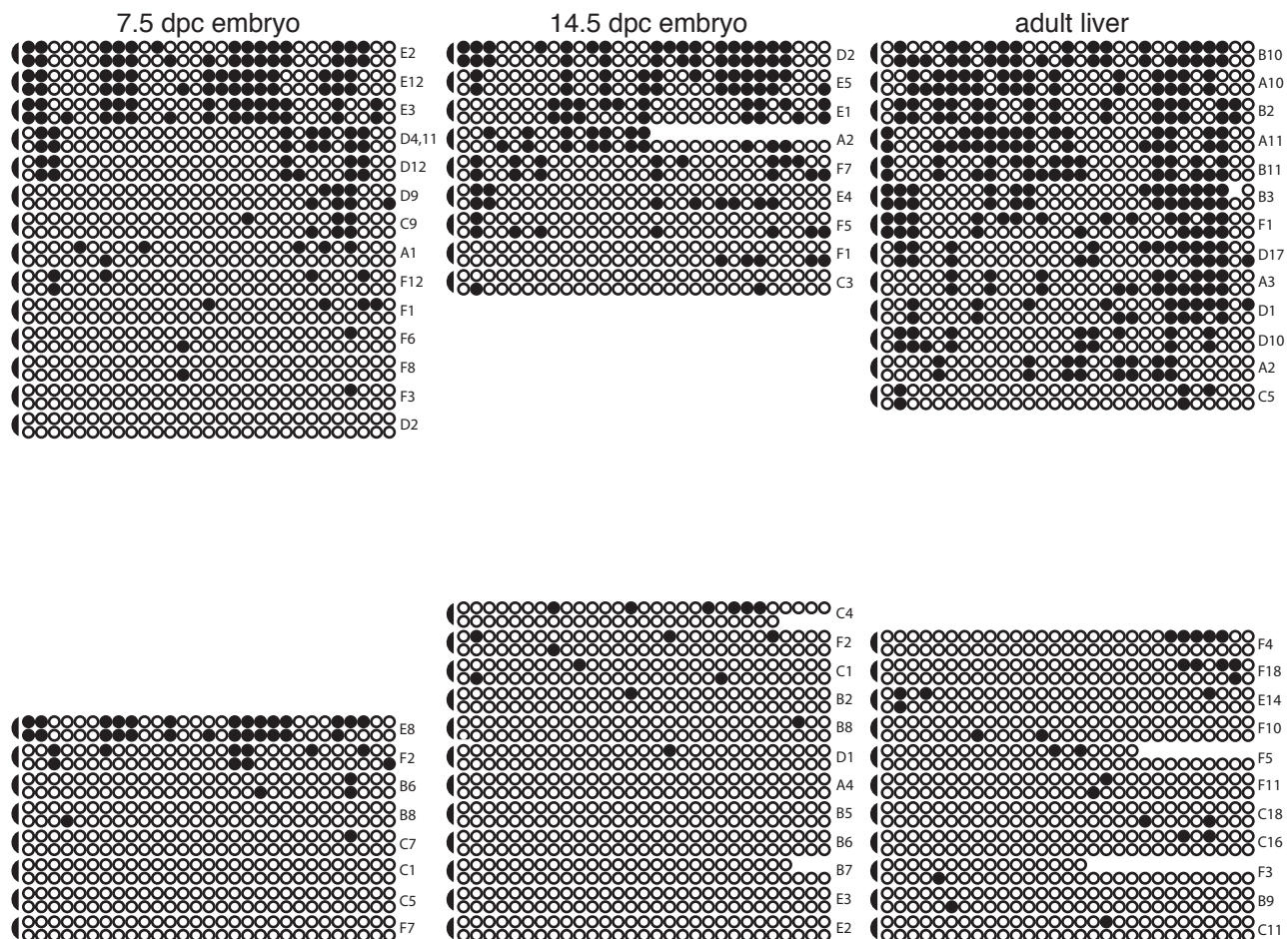

Supplement: Supplementary file 8 — Additional file 8: Figure S2. The maternally methylated secondary DMRs associated with Ndn and Peg12 display a high level of hemimethylation. (A) Methylation status at the Ndn DMR; F1 hybrid DNA derived from 14.5 dpc BxC embryos and adult brain. (B) Methylation status at the Peg12 DMR; F1 hybrid DNA derived from 7.5 and 14.5 dpc BxC embryos and adult BxC liver. Other details as described in Additional file 7: Figure S1. [file 13072_2019_309_MOESM8_ESM.pdf]

**A***H19* ICR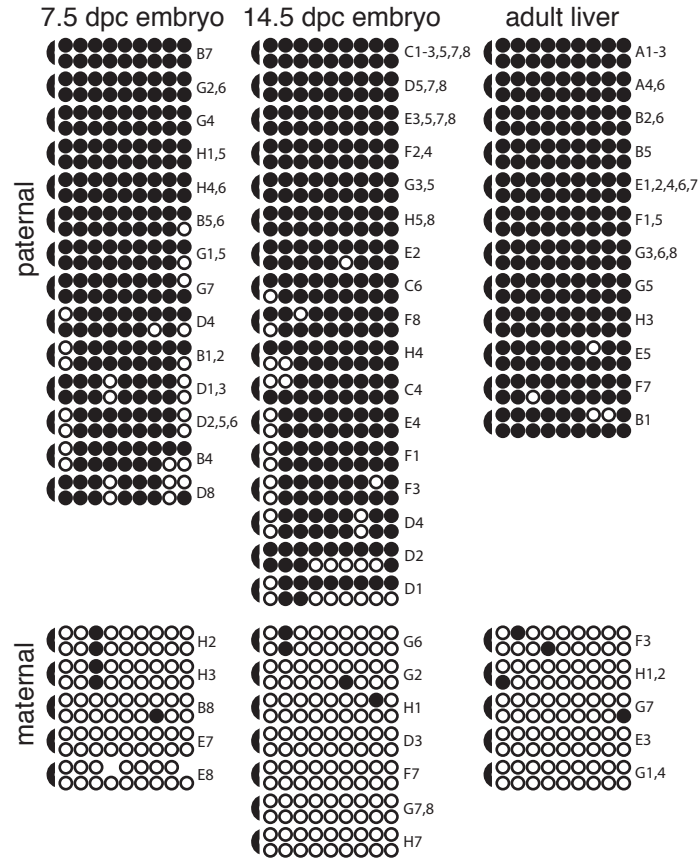**B***Snrpn* DMR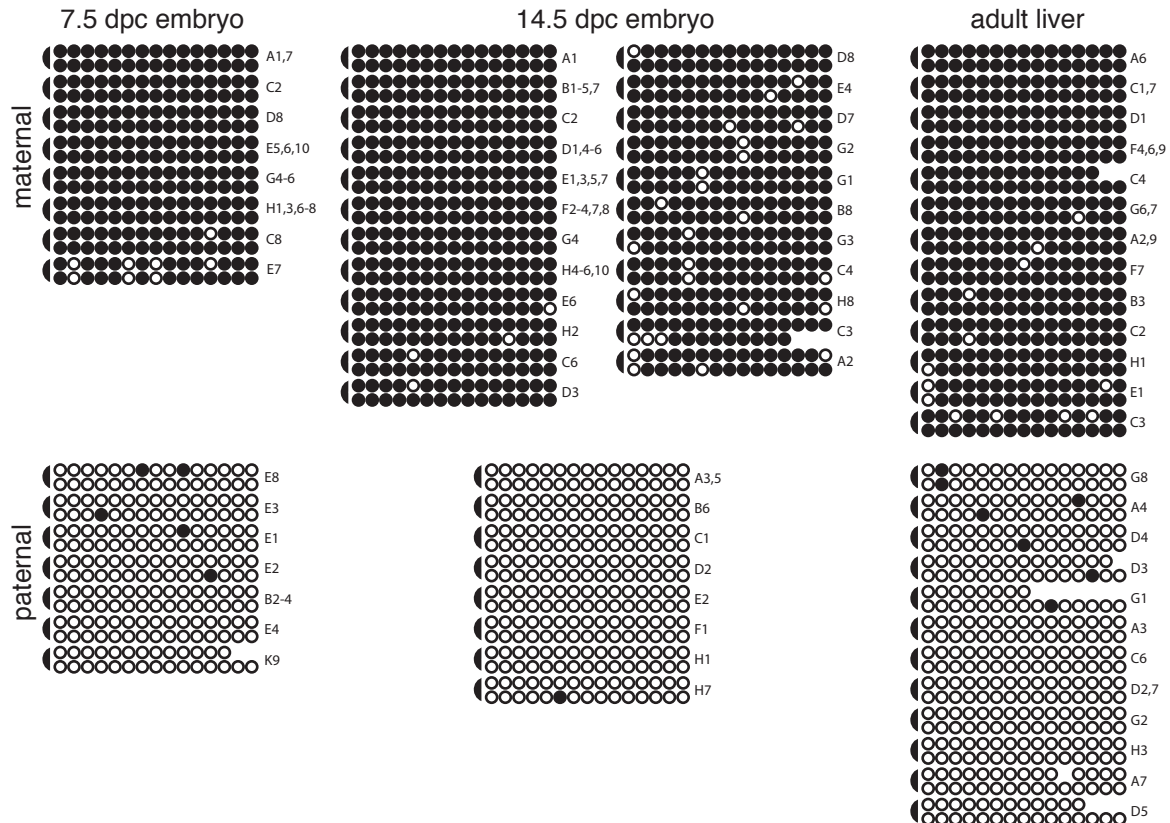

Supplement: Supplementary file 9 — Additional file 9: Figure S3. The primary DMRs associated with H19 and Snrpn display low levels of hemimethylation. Data shown are from DNA derived from 7.5 and 14.5 dpc BxC embryos and adult liver. Details as described in Additional file 7: Figure S1. [file 13072_2019_309_MOESM9_ESM.pdf]
