# Supplementary material for: Hemimethylation of CpG dyads is characteristic of secondary DMRs associated with imprinted loci and correlates with 5-hydroxymethylcytosine at paternally methylated sequences
Source: Epigenetics Chromatin. 2019 Oct 17;12:64. doi: 10.1186/s13072-019-0309-2 (PMC6796366; doi:10.1186/s13072-019-0309-2)
Supplement: Supplementary file 10 — Additional file 10: Figure S4. Reciprocal crosses illustrate that allele-specific methylation is dependent on parental origin, not strain. Data shown are from DNA derived from a 13.5 dpc CxB embryo. (A) Paternally and maternally methylated secondary DMRs. (B) Paternally and maternally methylated primary DMRs. Details as described in Additional file 7: Figure S1. [file 13072_2019_309_MOESM10_ESM.pdf]

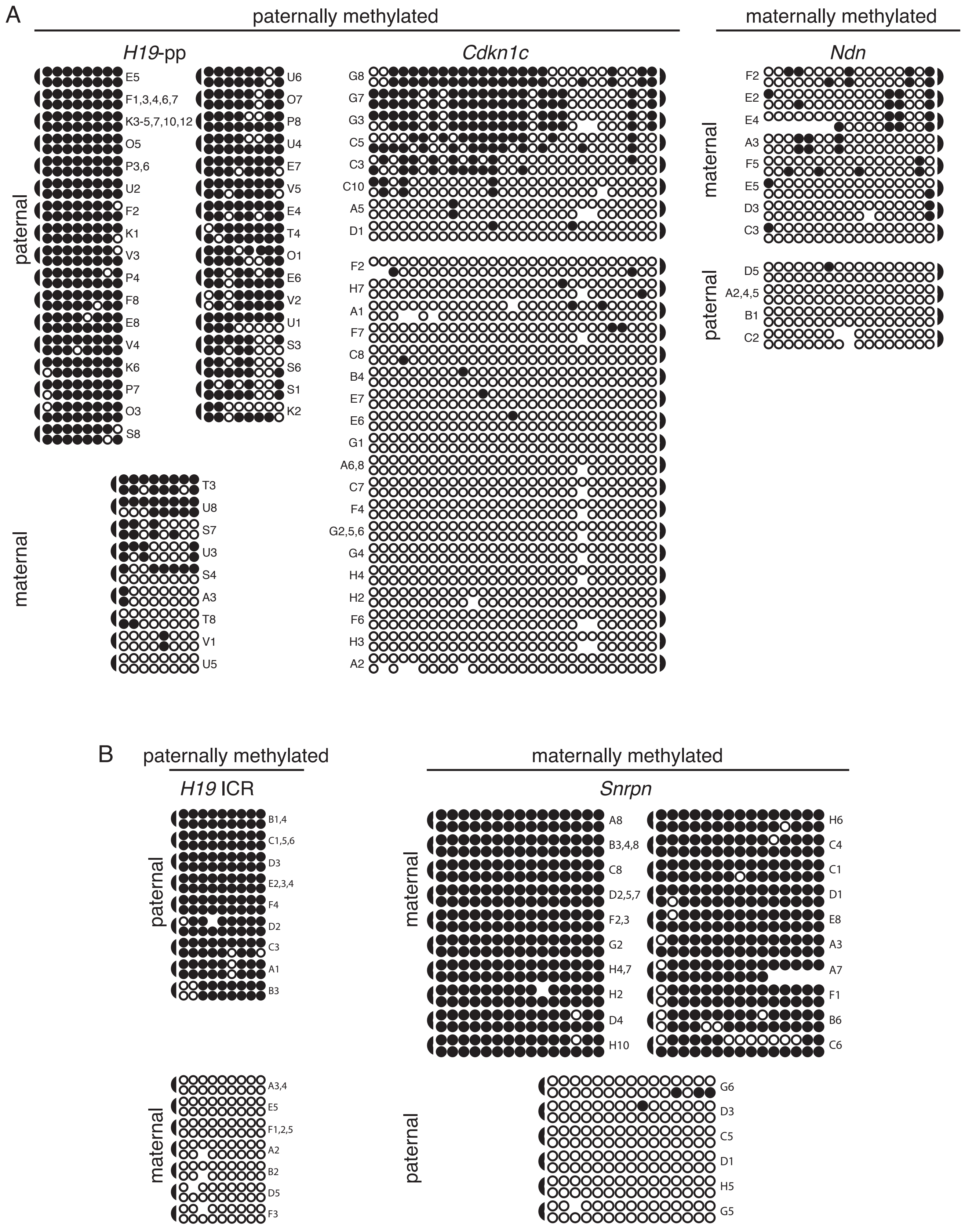
A
paternally methylated
maternally methylated
H19-pp
Cdkn1c
Ndn
paternal
maternal
E5
F1,3,4,6,7
K3-5,7,10,12
O5
P3,6
U2
F2
K1
V3
P4
F8
E8
V4
K6
P7
O3
S8
U6
O7
P8
U4
E7
V5
E4
T4
O1
E6
V2
U1
S3
S6
S1
K2
T3
U8
S7
U3
S4
A3
T8
V1
U5
G8
G7
G3
C5
C3
C10
A5
D1
F2
H7
A1
F7
C8
B4
E7
E6
G1
A6,8
C7
F4
G2,5,6
G4
H4
H2
F6
H3
A2
F2
E2
E4
A3
F5
E5
D3
C3
D5
A2,4,5
B1
C2
B
paternally methylated
maternally methylated
H19 ICR
Snrpn
B1,4
C1,5,6
D3
E2,3,4
F4
D2
C3
A1
B3
A3,4
E5
F1,2,5
A2
B2
D5
F3
A8
B3,4,8
C8
D2,5,7
F2,3
G2
H4,7
H2
D4
H10
H6
C4
C1
D1
E8
A3
A7
F1
B6
C6
G6
D3
C5
D1
H5
G5
